# Single-Walled Carbon Nanotubes Inhibit TRPC4-Mediated Muscarinic Cation Current in Mouse Ileal Myocytes

**DOI:** 10.3390/nano11123410

**Published:** 2021-12-16

**Authors:** Lina T. Al Kury, Dimitrios Papandreou, Vasyl V. Hurmach, Dariia O. Dryn, Mariia I. Melnyk, Maxim O. Platonov, Yuriy I. Prylutskyy, Uwe Ritter, Peter Scharff, Alexander V. Zholos

**Affiliations:** 1 College of Natural and Health Sciences, Zayed University, Abu Dhabi 144534, United Arab Emirates; Lina.Alkury@zu.ac.ae (L.T.A.K.); Dimitrios.Papandreou@zu.ac.ae (D.P.); 2 ESC “Institute of Biology and Medicine”, Taras Shevchenko National University of Kyiv, 64 Volodymyrska Str., 01601 Kyiv, Ukraine; gyrmach@gmail.com (V.V.H.); darinka.dr@gmail.com (D.O.D.); gribovamari@gmail.com (M.I.M.); prylut@ukr.net (Y.I.P.); 3 O.O. Bogomoletz Institute of Physiology, National Academy of Sciences of Ukraine, 4 Bogomoletz Str., 01024 Kyiv, Ukraine; 4 Institute of Pharmacology and Toxicology, National Academy of Medical Sciences of Ukraine, 14 Anton Tsedik Str., 03057 Kyiv, Ukraine; 5 Institute of Molecular Biology and Genetics, National Academy of Sciences of Ukraine, 150 Zabolotnogo Str., 03143 Kyiv, Ukraine; platon1971@gmail.com; 6 Institute of Chemistry and Biotechnology, Technical University of Ilmenau, 25 Weimarer Str., 98693 Ilmenau, Germany; uwe.ritter@tu-ilmenau.de (U.R.); peter.scharff@tu-ilmenau.de (P.S.)

**Keywords:** single-walled carbon nanotubes, TRPC4 channels, smooth muscle cell, gastrointestinal tract, computer simulation, patch clamp technique

## Abstract

Single-walled carbon nanotubes (SWCNTs) are characterized by a combination of rather unique physical and chemical properties, which makes them interesting biocompatible nanostructured materials for various applications, including in the biomedical field. SWCNTs are not inert carriers of drug molecules, as they may interact with various biological macromolecules, including ion channels. To investigate the mechanisms of the inhibitory effects of SWCNTs on the muscarinic receptor cation current (mI_CAT)_, induced by intracellular GTPγs (200 μM), in isolated mouse ileal myocytes, we have used the patch-clamp method in the whole-cell configuration. Here, we use molecular docking/molecular dynamics simulations and direct patch-clamp recordings of whole-cell currents to show that SWCNTs, purified and functionalized by carboxylation in water suspension containing single SWCNTs with a diameter of 0.5–1.5 nm, can inhibit mI_CAT_, which is mainly carried by TRPC4 cation channels in ileal smooth muscle cells, and is the main regulator of cholinergic excitation–contraction coupling in the small intestinal tract. This inhibition was voltage-independent and associated with a shortening of the mean open time of the channel. These results suggest that SWCNTs cause a direct blockage of the TRPC4 channel and may represent a novel class of TRPC4 modulators.

## 1. Introduction

Single-walled carbon nanotubes (SWCNTs) have become a hot research area because of their unique physico-chemical properties and prospects for various applications in nanotechnology. Owing to their nanosize, good biocompatibility, stability and high reactivity, SWCNTs can be widely used in biomedicine [1,2,3]. It has been reported that SWCNTs penetrate the cells via endocytosis-dependent and independent pathways [4,5,6]. They can affect neuronal activity [7], most likely at the level of ion channels [8]. SWCNTs have been defined as neuroprotectors [9] and effective substrates for the culturing of neurons [10]. As a nanoplatform, SWCNTs can be used for imaging and drug delivery [11,12]. We were the first to show that SWCNTs modulate cardiovascular control in rats [13]. However, the biomedical applications of SWCNTs are limited by their possible toxicity, so their biosafety is currently one of the most discussed issues. At present, information on SWCNT toxicity remains controversial [14,15,16]. The toxic effect of SWCNTs on normal cells and living systems is determined by the following factors: chemical composition, size, dose and exposure route [17,18]. Previously, we have reported that the systemic introduction of 0.1 mg/mL carboxylated SWCNTs (diameter of 0.5–1.5 nm) does not have any adverse effects is rats [19].

Earlier electrophysiological studies have shown that pristine SWCNTs (with diameter distributions peaking at approximately 0.9 and 1.3 nm) [20], as well as functionalized SWCNTs [8], can interact with various proteins, such as ion channels and receptors. In particular, they effectively block, in a dose-dependent manner, several different types of K^+^ channels heterologously expressed in mammalian cells. An electrical system based on a network of semiconducting nanotubes was developed to detect single ion channel activity by measuring the dynamic opening and closing of the individual ion channels using SWCNTs [8]. Moreover, SWCNTs were found to mimic some aspects of ion permeation via channel-forming proteins [21]. Such ion transport via SWCNTs (diameter of 1.2–2 nm) may even mimic some of the fundamental properties of ion channels, such as their voltage dependence [22].

We have previously characterized C_60_ fullerenes as novel inhibitors of large-conductance Ca^2+^-activated K^+^ channels in pulmonary artery myocytes [23] and the muscarinic receptor cation current, termed mI_CAT_, in ileal smooth muscle cells [24]. The latter current is mediated by both TRPC4 and TRPC6 proteins, with TRPC4 acting as its main molecular component [25]. Activated under physiological conditions by acetylcholine, mI_CAT_ is well recognized as the principal regulator of cholinergic excitation–contraction coupling in gastrointestinal smooth muscles [26,27]. Several inhibitors of this current are known, including ML204 [28], polyamines [29] and SK&F 96,365 [30]. In this study, we aimed to investigate the effects of SWCNTs on mI_CAT_ using both molecular modeling and direct patch-clamp recordings of whole-cell currents.

## 2. Materials and Methods

### 2.1. Preparation and Characterization of SWCNTs Water Suspension

SWCNTs were synthesized by means of the arc-discharge technique between two graphite electrodes in a He atmosphere (700 mbar) [5,31]. The anode was drilled and filled with catalytic powder (graphite, 1% Y_2_O_3_, 4.2% NiO). The arc-discharge was performed with a current of 150 A. The contaminants, such as amorphous carbon and metallic catalyst particles, were removed after treatment with boiling HCl (6 M) in a reflux condenser.

For the characterization of the SWCNTs, we applied high-resolution transmission electron microscopy (HRTEM, Tecnai 20 S-Twin, Beijing, China). According to the HRTEM micrographs, the diameter and the length of SWCNTs are 0.5–2 nm and 1–5 μm, respectively. Additionally, we also employed thermogravimetric analysis (TGA, Sartorius MC5, Wood Dale, IL, USA) in air before acid treatment. The TGA produced an unburned residue (catalyst particles) at T *=* 1323 K of 5 m% due to residual catalyst particles.

The functionalization (carboxylation) of SWCNTs was accomplished by HNO_3_ (3 M) treatment for 2 h at T = 373 K with the aim of improving their hydrophilicity. The chemical composition of the samples was characterized by X-ray photoelectron spectroscopy (XPS) measurements in normal emission using an ultra-high-vacuum (UHV) system equipped with a Physical Electronics 10-610 X-ray source [32]. The functionalized SWCNTs were suspended in distilled water by ultrasonication (UZDN-1 U42, Russia); 21 kHz; 0.68 A, processing time of 90 s at constant heat removal) allowing dissolution of some SWCNTs in water. The obtained suspension was filtered out by means of a membrane filter (pore size was 1.2 μm). The maximum concentration of SWCNTs in water (0.1 mg/mL) was determined (Analytik Jena TOC Analyser multi N/C 3100, Jena, Germany) as the concentration of total organic carbon in the water suspension.

The purity of prepared SWCNT water suspension was determined by high-performance liquid chromatography (Jasco PU-2086, Tokyo, Japan) and GC/MS using standard programs. Insoluble impurities were determined by ultracentrifugation. The insoluble impurities in the prepared SWCNT water suspension were found to be less than 1 μg/mL. The SWCNT water suspension was stable for about three months at T = 283 K [33].

In order to characterize the composition of the prepared SWCNTs water suspension, atomic force microscopy (AFM) was performed. According to the AFM results, the majority of SWCNTs in water suspension (0.1 mg/mL) appear as aggregates (bundles) with a height of up to 40 nm [5]. However, non-aggregated, i.e., single, SWCNTs with a diameter of 0.5–1.5 nm, which can interact with the TRPC4 channel pore and are consistent with the selected computed molecular models (see below), were also present [19].

### 2.2. Molecular Docking

Prior to molecular docking, it was taken into consideration that the diameter of tested SWCNTs was in the range of 0.4–20 nm. Therefore, all possible SWCNTs were generated using a CHARMM-GUI nanomaterial modeler [34,35,36,37]. Based on the structural analysis and TRPC4 location in the lipid membrane according to PDBTM [38] and PMM [39], we simulated SWCNT interaction with the TRPC4 channel pore.

Molecular docking was performed by utilizing the flexible SWCNT and rigid TRPC4 channel. A systematic docking algorithm was used (SDOCK+) [40], implemented in the QXP package [41] (the method demonstrates all possible conformations of the studied structures with a minimum RMSD value [40]). In total, 300 possible “SWCNT-TRPC4” complexes were generated for all considered SWCNTs. Then, the 10 best complexes were selected using QXP scoring functions [41] for the next stages (visual inspection). The optimal structure of the studied “SWCNT-TRPC4” complexes was determined by the following basic criteria: (1) the number of hydrogen bonds; (2) the area of the contact surfaces of the protein and ligand; (3) the distance between the protein and ligand; (4) the energy characteristics of the binding in the formed complex.

### 2.3. Molecular Dynamics (MD) Simulation

**Figure 1:**
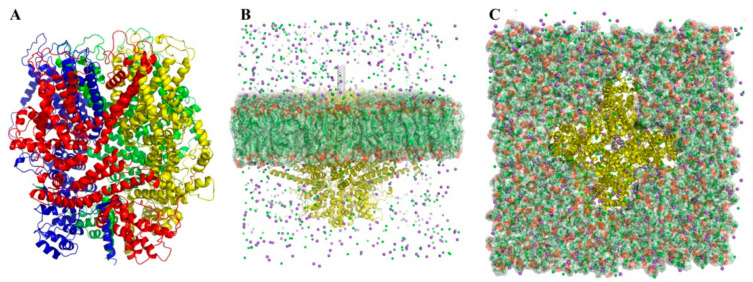
(**A**) Molecular model of receptor-operated TRPC4 cation channel (PDB ID 5Z96) with its four subunits shown by different colors: subunit A—red, subunit B—blue, subunit C—green, and subunit D—yellow. (**B**) Profile view of TRPC4 channel. (**C**) Top view of TRPC4 channel. In both (**B**) and (**C**), the plasma membrane is highlighted in green, and an example of single-walled carbon nanotube (SWCNT) docked into the TRPC4 channel pore is shown in grey.

CHARMM-GUI Membrane Builder [42,43,44,45,46,47] was used for POPC (palmitoyl oleoyl phosphatidylcholine) membrane creation by adding of missed amino acids to the TRPC4 channel (Q9QUQ5 sequence was used) and embedding the TRPC4 channel into 700 POPC molecules, according to the PMM server [39] (Figure 1). The POPC membrane and TRPC4 channel modeled in the CHARMM-GUI Membrane Builder were energy-minimized and equilibrated in an environment similar to cellular content (water box with 0.15 M Na^+^ and Cl^−^ ions concentration). The protein was protonated according to the built-in functions of gromacs 2020. All calculations were performed using gromacs 2020 (http://www.gromacs.org/, last accessed date 11 June 2021) in a force field Charmm36 [48] at 300 K and at constant atmospheric pressure. Finally, the MD simulation lasted 50 ns.

### 2.4. Cell Isolation

All electrophysiological studies were performed using freshly isolated smooth muscle cells of murine small intestine. For the study, 3-month-old BALB/c male mice were used. All animal studies using BALB/c mice were carried out in accordance with the recommendations of the EU Directive 2010/63 on the protection of animals used for scientific purposes and approved by the Institutional Ethics Committee (No. 04/20).

Mice were humanely euthanized by CO_2_ asphyxia, then the abdominal cavity was opened and the longitudinal smooth muscle layer of the ileum was isolated and placed into modified normal Krebs solution of the following composition (in mM): 120 NaCl, 12 glucose, 10 HEPES, 6 KCl, 2.5 CaCl_2_, 1.2 MgCl_2_, pH adjusted to 7.4 with NaOH. The tissue was cut into small 1 mm pieces in Ca, Mg-free Krebs solution (in mM: 120 NaCl, 12 glucose, 10 HEPES, 6 KCl, pH adjusted to 7.4 with NaOH) and enzymatically treated in a mixture of collagenase type 1A, soybean trypsin inhibitor and bovine serum albumin (all reagents were used at 1 mg/mL) at 37 °C for 17 min. Then, tissue pieces were washed out three times from enzymes and mechanically triturated with a heat-polished glass Pasteur pipette until a cloudy appearance was obtained in the solution. The cell suspension was stored at 5–7 °C for 6–8 h after cell isolation.

### 2.5. Patch Clamp Recordings

Membrane currents were recorded via the patch-clamp techniques in a whole-cell configuration using an Axopatch 200B amplifier (Molecular Devices, Sunnyvale, CA, USA) at room temperature (22–25 °C). The protocols of voltage pulses were generated and data were recorded using a Digidata 1322A interfaced to a computer running the pClamp 8 software (Molecular Devices, Sunnyvale, CA, USA).

Patch-pipettes were fabricated from borosilicate glass (1.5 mm OD, 0.86 mm ID; Sutter Instrument, Novato, CA, USA) using a P-97 Flaming/Brown micropipette puller (Sutter Instrument, Novato, CA, USA) with a resistance of 3–3.5 MOhm when filled with the intracellular solution.

Before the current recordings, the cells were kept in normal Krebs solution, while for mI_CAT_ recordings, the bath solution was replaced with a Cs^+^-containing solution (in mM: 120 CsCl, 12 glucose, 10 HEPES, pH adjusted to 7.4 with CsOH). The pipette solution (in mM: 80 CsCl, 1 MgATP, 5 creatine, 5 glucose, 10 BAPTA, 10 HEPES, 4.6 CaCl_2_, pH adjusted to 7.4 with CsOH) contained 200 μM GTPγS, which activates G-proteins directly and thus initiates mI_CAT_, bypassing the muscarinic receptors.

Whole-cell recordings were filtered at 2 kHz and sampled at 10 kHz for analysis. Series resistance was compensated for by ~40%. The steady-state current–voltage relationships of mI_CAT_ were measured by slow 6 s voltage ramps from 80 to −120 mV, which were applied every 30 s. The holding potential was −40 mV.

### 2.6. Chemicals

All reagents and chemicals for electrophysiological studies were purchased from Sigma Chemical (St. Louis, MO, USA).

### 2.7. Data and Statistical Analysis

Patch-clamp data were analyzed and plotted using Clampfit 8 (Molecular Devices, Sunnyvale, CA, USA) and OriginPro 2021 software (OriginLab Corporation, Northampton, MA, USA). Data are presented as means ± SEM (standard error of the mean) with *n* indicating the number of cells used for a particular set of measurements. The Kolmogorov–Smirnov normality test was used, while differences between two groups were evaluated using Student’s paired *t*-test and considered significant at *p* < 0.05.

## 3. Results

### 3.1. Modeling of SWCNT Binding to TRPC4 Channel

To evaluate the possibility of SWCNT binding to the TRPC4 channel, we applied computer simulation techniques, namely, molecular docking and molecular dynamics (MD). Molecular docking is among the most frequently used numerical techniques, due to its ability to predict the binding-conformation of small molecule ligands to the appropriate target binding site [49]. MD simulation is also one of the most frequently used techniques to evaluate the stability of the “ligand-protein (target)” molecular system [23,50].

**Figure 2:**
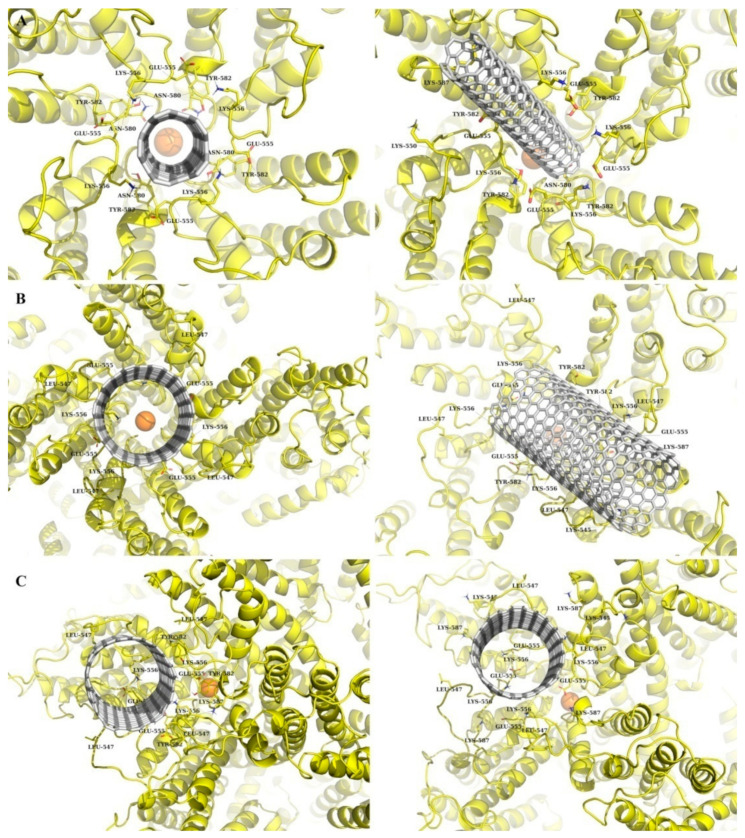
Results of molecular docking (left) and MD simulation (right) for SWCNTs: (**A**) SWCNT_1 model; (**B**) SWCNT_2 model, and (**C**) SWCNT_3 model. The TRPC4 channel is shown in yellow and SWCNT in grey.

The molecular model of the homotetrameric receptor-operated TRPC4 cation channel (mouse TRPC4 ion channel, PDB ID 5Z96) is shown in Figure 1. As a result of molecular docking, three optimal “SWCNT-TRPC4” complexes were selected (Figure 2, left). The first one is based on an SWCNT with a diameter of ~0.7 nm (SWCNT_1 model) (Figure 2A, left). In this case, SWCNT perpendicularly interacts within the plane of the TRPC4 channel pore and the palmitoyl oleoyl phosphatidylcholine (POPC) bilayer, and also undertakes similar interactions with each subunit of the TRPC4 channel (see Figure 1). Specifically, these interactions include the following: steric interaction with Asn 580 and Glu 555, cation-π-with Lys 556, and the possibility of t-stacking with Tyr 582. In the second model, an SWCNT (SWCNT_2 model) with a diameter of ~1.5 nm was used (Figure 2B, left). As in the first model, this SWCNT also binds perpendicularly to the plane of the TRPC4 channel and the POPC bilayer. However, an SWCNT cannot sink into the channel pore, and interacts only with its top part. As a result, the interaction between TRPC4 and SWCNT is weak: it is just a steric interaction with Leu 547, Lys 556 and Glu 555. Finally, in the last model, an SWCNT with a diameter of 1.5 nm was used (SWCNT_3 model shown in Figure 2C, left). In this case, an SWCNT does not interact similarly with each subunit of the TRPC4 channel. This SWCNT lies on its side, and one part partially sinks into the TRPC4 channel pore. Such a binding mode results in binding to the B subunit of the TRPC4 channel (see Figure 1), mostly via cation-π, of Lys 556 and Lys 587, and sterically with Glu 555, Tyr 582 and Leu 547 interactions.

To obtain more precise results, MD simulation was performed for the “SWCNT-TRPC4” complexes selected based on the molecular docking findings. The results are shown in Figure 2 (right). In all cases, SWCNTs can stably bind to the TRPC4 channel pore. Moreover, SWCNTs do not contact each subunit of the TRPC4 channel in the same manner as in the case of molecular docking. According to the MD simulation, the absorption of SWCNTs by the TRPC4 extracellular loops located near the pore was observed (especially in the SWCNT_1 and SWCNT_2 models). Furthermore, it was found that the SWCNTs, during interaction with the TRPC4 channel, could rotate and partially interact with different outer membrane residues of the pore in a non-symmetrical manner. Thus, in the case of the SWCNT_1 model, SWCNT was characterized by large displacement with a root–mean–square deviation (RMSD; value of 1.34 nm), and had strong interactions with Lys 556, Lys 587, Lys 550, Glu 555, Tyr 582 and Asn 580 (Figure 2A, right). In the case of the SWCNT_2 model, a similar situation was observed: SWCNT displacement was 0.97 nm; the main binding residues were almost the same, except for Lys 545, Lys 587, Leu 547 and Asn 580 (Figure 2B, right). Finally, in the case of the SWCNT_3 model, the RMSD value was about 0.5 nm, and in this case, SWCNT interacted with Glu 555, Lys 587, Lys 545, Lys 587, Lys 556, Glu 555 and Leu 547 (Figure 2C, right).

**Figure 3:**
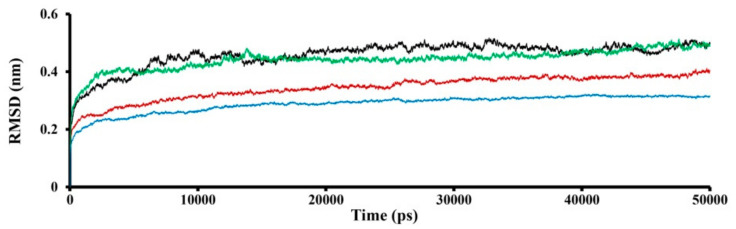
The root–mean–square deviation (RMSD) trajectories of TRPC4 channel alone (in red) and in complex with SWCNTs: SWCNT_1 model is shown in black, SWCNT_2 model in green and SWCNT_3 model in blue.

According to the RMSD movement of the obtained complexes, the SWCNT_1 and SWCNT_2 models are more flexible than the unbound TRPC4 channel and the SWCNT_3 model (Figure 3).

**Figure 4:**
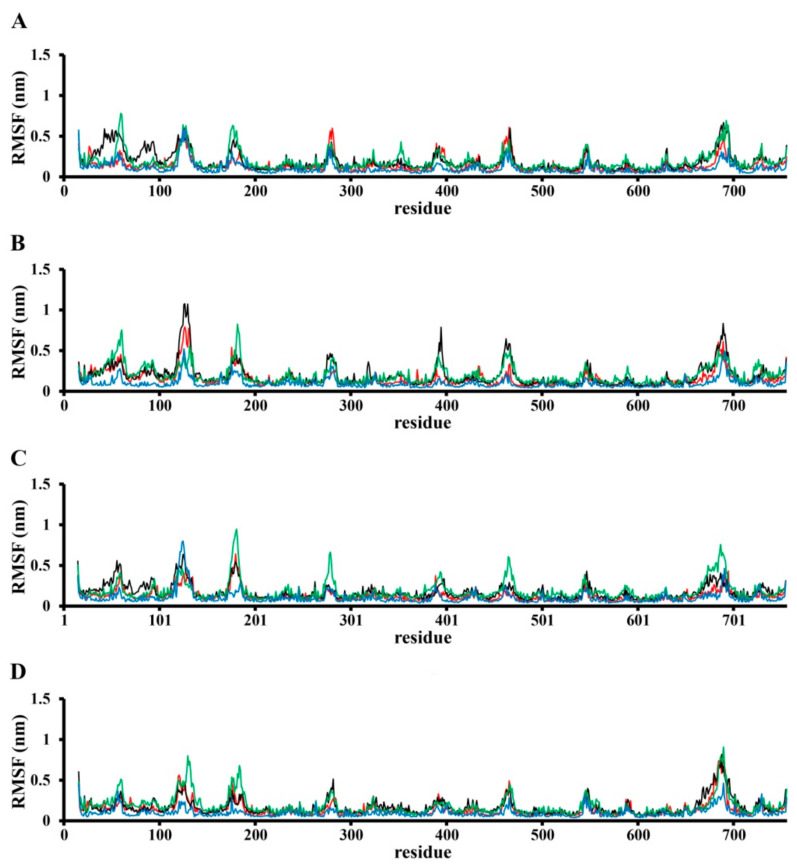
The root–mean–square fluctuation (RMSF) profile of the TRPC4 channel alone (in red; (**A**–**D**) subunits) and in complex with SWCNTs: the SWCNT_1 model is shown in black, SWCNT_2 model in green and SWCNT_3 model in blue.

The RMSF (root–mean–square fluctuation) profile analysis (Figure 4) revealed that binding with SWCNT affects the whole TRPC4 channel structure. The fluctuation of the TRPC4 channel pore in the binding region and nearby parts was affected differently by each SWCNT in each binding model. For example, pore flexibility (residues 545–560 and 578–587) allows for binding in the SWCNT_1 model that is almost the same as that with the unbound TRPC4 channel in the SWCNT_2 model, but this binding is somewhat stronger compared to the SWCNT_3 model (Figure 4). The results in other parts of the TRPC4 channel, especially the intracellular section, were surprising. For example, the movements of residues 163–186 were different in all simulation cases (Figure 4).

**Figure 5:**
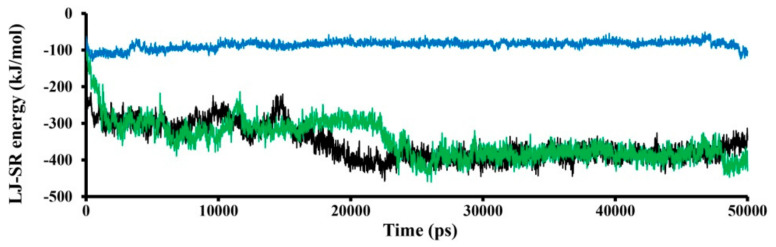
The Lennard–Jones short range (LJ-SR) binding energy between the TRPC4 channel and SWCNTs: SWCNT_1 model is shown in black, SWCNT_2 model in green and SWCNT_3 model in blue.

The LJ-SR (Lennard–Jones short range) energy computed in this simulation is better in the case of the SWCNT_2 binding model (Figure 5). Clearly, the interacting surfaces between the TRPC4 channel and SWCNT_2 model are larger compared to the SWCNT_1 model. The LJ-SR energy of the SWCNT_3 model is about −100 kJ/mol, and it does not change over time. In the case of the SWCNT_1 and SWCNT_2 models, the calculated LJ-SR energy is about −300 and −400 kJ/mol in the first (0–25,000 ps) and second phases of calculation (2500–50,000 ps), respectively.

In summary, we can conclude that all considered SWCNTs can interact with the TRPC4 channel by forming one or another stable bond.

### 3.2. Inhibitory Action of SWCNT on mI_CAT_ in Ileal Myocytes

**Figure 6:**
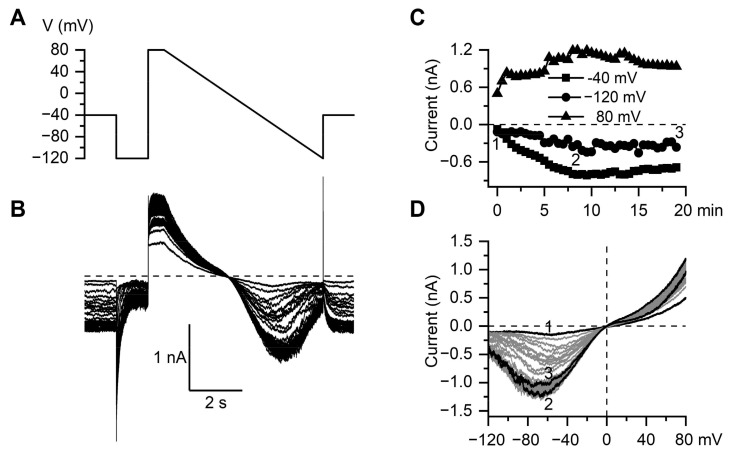
Characterization of GTPγS-induced mI_CAT_ in mouse ileal myocytes. (**A**) Voltage protocol and (**B**) corresponding representative superimposed linear leak-corrected current traces of the cationic current activated by GTPγS infusion. (**C**) Time course of GTPγS-induced mI_CAT_ development at the three different test potentials (−40, −120 and 80 mV) as indicated by different symbols. These represent mean current amplitudes at the holding potential of −40 mV and during the last 200 ms of voltage steps to −120 and 80 mV. (**D**) Corresponding superimposed current-voltage relations of mI_CAT_ measured by the slow (6 s duration) voltage ramp from 80 to −120 mV (**A**). Shown in black are three I-V curves measured immediately after membrane break-through (**A**), at the peak response to GTPγS (**B**) and at the end of the experiment (**C**), as indicated by the same letters in panel (**C**).

In ileal myocytes, the TRPC4 receptor-operated cation channels are activated in synergy with M2 and M3 receptors, which are differentially coupled to G_q/11_ and G_i/o_ proteins [51,52]. Although adding GTP to the pipette solution counteracts the desensitization of the response to some extent [53], the current is most stable when activated by the hydrolysis-resistant GTP analog GTPγS, which interacts with G-proteins directly. Figure 6A illustrates the voltage protocol used in our experiments. For a complete assessment of both the kinetics and voltage dependence of mI_CAT_, a combination of voltage steps and a slow (6 s duration) voltage ramp was applied, with the holding potential of −40 mV with an interval of 30 s starting shortly (20–30 s, time needed to adjust membrane capacitance and series resistance compensations) after break-through with a patch pipette containing GTPγS (200 μM). mI_CAT_ was effectively isolated using symmetrical Cs^+^-containing (125 mM) solutions, with a strong buffering intracellular free Ca^2+^ concentration of 100 nM employed to avoid the complexity of its calcium-dependent regulation, such as current fluctuations concomitant with intracellular Ca^2+^ oscillations [54,55]. Under these conditions, the current slowly increased in size to reach a peak amplitude in about 5–7 min, along with gradual accumulations in the cells of G-proteins that were spontaneously and irreversibly activated by GTPγS (Figure 6A,C). Figure 6D illustrates the corresponding superimposed steady-state current–voltage (I-V) relationships of mI_CAT_ obtained by slow voltage ramps. The steady-state I-V relationship was doubly rectifying around the reversal potential (E_REV_ close to 0 mV) and U-shaped at negative potentials, which is typical for mI_CAT_. It can be seen that very little run-down of the current occurred during the time course of the experiment under control conditions (Figure 6D, traces marked as 2 and 3).

**Figure 7:**
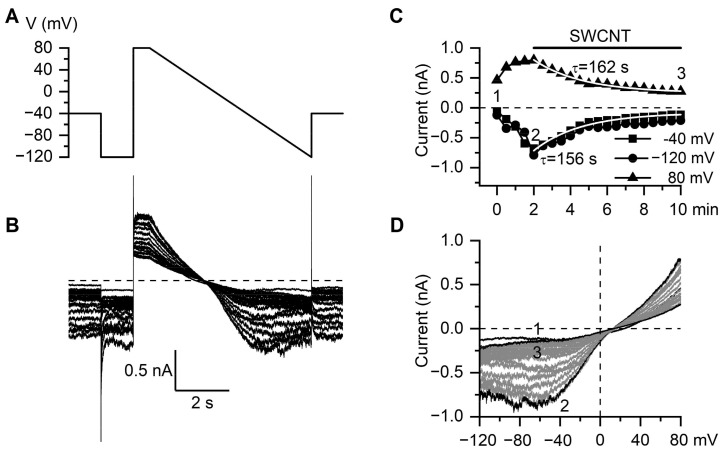
SWCNTs inhibit the muscarinic cation current via TRPC4 channels. (**A**) Voltage protocol and (**B**) corresponding representative superimposed current traces of the cationic current activated by GTPγS infusion and then suppressed by SWCNTs. (**C**) Time course of mI_CAT_ development in response to GTPγS followed by its time-dependent inhibition as assessed at three different test potentials (−40, −120 and 80 mV). Superimposed white lines show that, after SWCNT application, mI_CAT_ mono-exponentially declined with similar time constants both at negative and positive potentials. (**D**) Corresponding superimposed current–voltage relations of mI_CAT_ measured by the slow (6 s duration) voltage ramp from 80 to −120 mV (**A**). Shown in black are three I-V curves measured immediately after membrane break-through (**A**), at the peak response to GTPγS (**B**) and at the steady-state current inhibition by SWCNTs (**C**), as indicated by the same letters in panel (**C**).

The application of SWCNTs (10 μg/mL) after the GTPγS-induced mI_CAT_ reached its peak amplitude markedly inhibited the current (Figure 7). The inhibition developed mono-exponentially with time constants of 156 and 162 s at −40 and 80 mV, respectively, as shown by the superimposed white lines in Figure 7C. The mean time constant of mI_CAT_ inhibition by SWCNTs at 80 mV was 180 ± 31 s (min = 109.2 s; median = 162.6 s; max = 267.6 s; n = 5).

**Figure 8:**
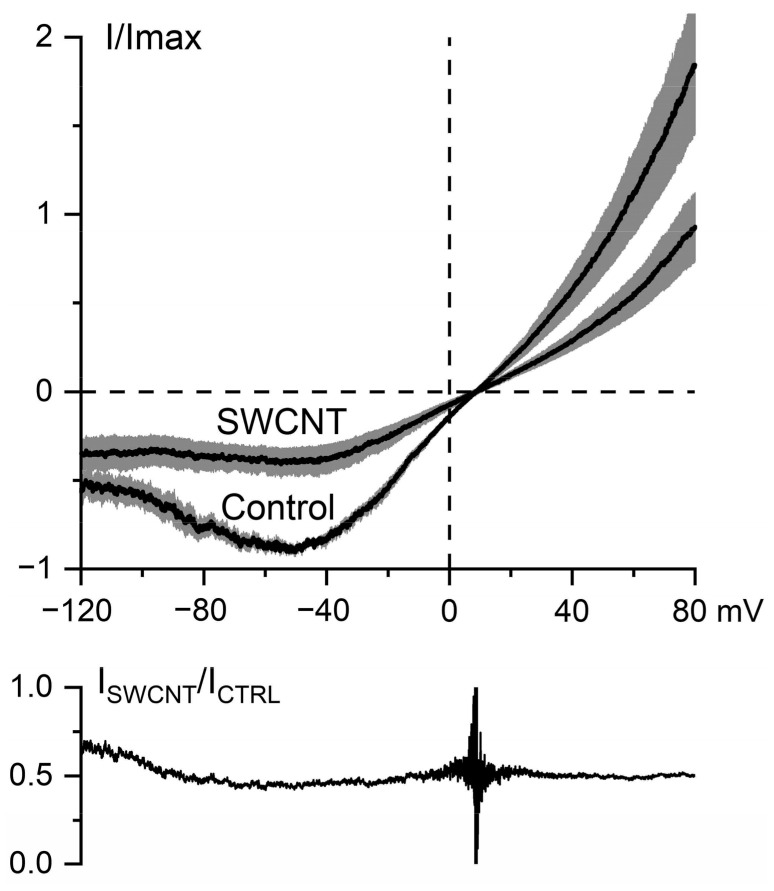
SWCNTs inhibit the muscarinic cation current via TRPC4 channels in a voltage-independent manner. Top, mean normalized I-V curves of GTPγS-induced currents at maximal current activation in the control compared to those during mICAT steady-state inhibition by SWCNTs applied at 10 µg/mL. The grey bands represent the SEM values (n = 5). Bottom, ratio of current amplitude in the presence of SWCNTs to that in control plotted vs. test potential.

Figure 8 shows the mean normalized I-V curves of GTPγS-induced mI_CAT_ under control conditions and after current inhibition by SWCNTs (10 μg/mL). For quantification of the inhibitory effect, the maximal inward current in each cell under control conditions was normalized as 1.0, so that each cell could serve as its own control for the inhibitory effect of SWCNTs. Its mean value was −650.4 ± 146.1 pA, decreasing to −269.2 ± 84.0 pA after SWCNTs application (paired *t*-test, two-tail *p* value of 0.03; n = 5). Notably, mI_CAT_ was inhibited to a similar extent in the whole range of membrane potentials, as the ratio of currents in Figure 8 (bottom panel) shows. It should be noted that somewhat higher values of the ratio at potentials below −90 mV are related to the smaller mI_CAT_ due to the voltage-dependent deactivation of TRPC4 channels together with the relatively larger nonspecific currents at these potentials.

Considering that the rate of current deactivation with the voltage step from −40 mV to −120 mV (Figure 7A,B) reflects the mean channel open time at −40 mV^55^, additional analysis was performed by fitting current decline at −120 mV via the single exponential function. The mean deactivation time constant was 35.2 ± 3.3 ms at peak current activation, as assessed by GTPγS application under control conditions, reducing to 18.1 ± 3.1 ms 6 min after SWCNTs application (paired *t*-test, two-tail *p* value of 0.018; n = 5). These findings suggest that SWCNTs inhibit the TRPC4 channel by shortening its open state, consistently with the results of molecular docking and MD simulations (Figure 1, Figure 2, Figure 3, Figure 4 and Figure 5).

## 4. Discussion

Members of the superfamily of Ca^2+^-permeable TRP channels are expressed in almost all cells of the body, where they perform different important functions, ranging from regulation of membrane potential and calcium signaling to signal transduction determining cell growth, proliferation and death. In mammals, this superfamily of cation channels consists of 28 members subdivided into 6 subfamilies based on their structural similarities, namely, TRPC (canonical), TRPV (vanilloid), TRPM (melastatin), TRPA (ankyrin), TRPP (polycystin), and TRPML (mucolipin) [56]. Notwithstanding substantial differences in their intracellular carboxy- and amino-terminus, all TRPs have similar membrane topologies, including six transmembrane domains (S1–S6), with the pores of the channel formed by S5 and S6 domains, as well as P-loop. Within this superfamily, seven members of the canonical TRPC subfamily (TRPC1-TRPC7) are all calcium-permeable receptor-operated channels, which are commonly activated downstream of phospholipase C activation, and carry out diverse regulatory functions in the nervous system, heart, lung, vasculature, immune cells and gastrointestinal tract [56].

In the present study, we focused on TRPC4 channels, specifically on their role as the principal molecular component of mI_CAT_ [25]. Other functions of TRPC4, as revealed by the knockdown of TRPC4 expression in native cells and studies of TRPC4 knockout mice, include the regulation of endothelial permeability, vascular tone, and neurotransmitter release. Moreover, TRPC4 channels have been implicated in epileptogenesis, excitotoxicity and urinary bladder overreactivity. Thus, TRPC4 channels are believed to be promising molecular targets for pharmacological interventions in treating several disease states [57]. However, presently, there are only two known potent and specific blockers of TRPC4 channels, ML204 [28], and the more recently developed synthetic compound Pico145 [58]. Although we have previously documented the inhibitory action of C_60_ fullerenes on mI_CAT_ in ileal myocytes [24], our biophysical analysis has shown that C_60_ fullerenes are unlikely to be direct channel blockers of TRPC4; rather, they are likely to accumulate in the membrane and disrupt G-protein mediated signaling, leading to channel opening, thus acting as channel gating modifiers. In contrast, the inhibition of mI_CAT_ by SWCNTs was shown to be voltage-independent (Figure 8), thus evidencing that molecular interactions with channel proteins occurred outside of the membrane voltage field, e.g., within the extracellular part of the channel. This notion is generally consistent with our in silico molecular modeling predictions. Depending on the size of SWCNT nanoparticles, three optimal binding models were considered, all of which demonstrated stable binding. In the case of SWCNTs of 0.7–1.5 nm size, interactions occurred within the plane of the TRPC4 channel pore and the POPC bilayer (our SWCNT_1 and SWCNT_2 models). The SWCNT_3 model predicted that a SWCNT could even partially sink into the TRPC4 channel pore. Thus, molecular modeling predicts channel pore hindrance for cation entry into the channel, the result of which is generally consistent with our experimental findings showing voltage-independent inhibition and some shortening of the mean open time of the channel. Future mutagenesis experiments can address the specific roles of certain amino acid residues in SWCNTs binding to definitively show the SWCNTs–TRPC4 interaction. However, such experiments have certain limitations/drawbacks. First, channel mutagenesis will necessitate the heterologous expression of the mutant channels, while the main purpose and advantage of our study was to characterize SWCNTs’ action on native mI_CAT_. Second, interacting surfaces between SWCNTs and the TRPC4 protein can be quite large, as up to seven amino acid residues in each of the four pore-forming subunits are involved, according to our molecular docking and MD simulations (Figure 2). Third, the mutagenesis of amino acid residues at a channel’s pore is likely to have multiple effects on ion permeation and channel gating; some may even render the channel inactive, thus requiring additional controls.

SWCNTs, with a diameter of about 1.3 nm, have been previously shown to inhibit, in the same concentration range, several types of K^+^ channels expressed in CHO cells, including HERG, but not chloride CLC3 channels [20]. Taken together with our present results, a more general picture now emerges that these nanostructured materials may represent a new class of general cation channel blockers. In contrast, we found that C_60_ fullerenes inhibited TRPC4 and large-conductance Ca^2+^-activated K^+^ channels, but not voltage-gated K^+^ channels [23,24]. Thus, as ion channel blockers, compared to C_60_ fullerenes, SWCNTs are less discriminative, and considering their HERG effects [20], they may even be cardiotoxic.

While it remains to be seen whether or not SWCNTs may be useful for the correction of intestinal motility disorders associated with visceral cholinergic smooth muscle hyperactivity, such as irritable bowel syndrome, our study is of particular interest in connection with one of the proposed biomedical applications of carbon nanotubes, namely, as carriers for the delivery of drugs, especially anti-cancer drugs. Since Cheung et al. [59] have demonstrated the involvement of TRPC4 and TRPC5 channels in adverse reactions to the potent cancer cell-specific cytotoxic agent (-)-Englerin A, situations may be envisaged wherein TRPC4 channel inhibition by SWCNTs carrying anti-cancer drugs may even be beneficial. However, the full assessment of such potential benefits requires additional studies, including evaluations of the concentration dependence and reversibility of the inhibitory action of SWCNTs on mI_CAT_.

## Data Availability

Datasets analyzed or generated during the study are available from the corresponding author upon reasonably justified request.

## Abbreviations

SWCNTs	single-walled carbon nanotubes
M receptors	muscarinic receptors
mI_CAT_	muscarinic receptor cation current
GTPγS	guanosine 5′-O-[gamma-thio]triphosphate
TRPC4	transient receptor potential cation channel subfamily C member 4
SM	smooth muscles
HRTEM	High-resolution transmission electron microscopy
TGA	thermogravimetric analysis
AFM	atomic force microscopy
SDOCK	systematic docking algorithm
POPC	palmitoyl oleoyl phosphatidylcholine
MD	molecular dynamics
RMSD	root–mean–square deviation
RMSF	root–mean–square fluctuation
LJ-SR energy	Lennard–Jones short-range energy
E_REV_	reversal potential
